# Thermal Stability and Rheological Properties of Epoxidized Natural Rubber-Based Magnetorheological Elastomer

**DOI:** 10.3390/ijms20030746

**Published:** 2019-02-10

**Authors:** Nurul Azhani Yunus, Saiful Amri Mazlan, Ubaidillah, Siti Aishah Abdul Aziz, Salihah Tan Shilan, Nurul Ain Abdul Wahab

**Affiliations:** 1 Department of Mechanical Engineering, Universiti Teknologi PETRONAS, 32610 Bandar Seri Iskandar, Malaysia; 2 Advanced Vehicle System Laboratory, Malaysia–Japan International Institute of Technology, Universiti Teknologi Malaysia, Jalan Sultan Yahya Petra, 54100 Kuala Lumpur, Malaysia; amri.kl@utm.my (S.A.M.); aishah118@gmail.com (S.A.A.A.); salihahtsl@yahoo.com (S.T.S.); ain_iman@yahoo.com (N.A.A.W.); 3 Mechanical Engineering Department, Faculty of Engineering, Universitas Sebelas Maret, Jl. Ir. Sutami 36A, Kentingan, Surakarta, 57126 Surakarta, Indonesia; ubaidillah@uns.ac.id; 4 National Center for Sustainable Transportation Technology (NCSTT), 40132 Bandung, Indonesia

**Keywords:** magnetorheological elastomers, epoxidized natural rubber, thermal properties, thermal stability, rheological properties

## Abstract

Determination of the thermal characteristics and temperature-dependent rheological properties of the magnetorheological elastomers (MREs) is of paramount importance particularly with regards to MRE applications. Hitherto, a paucity of temperature dependent analysis has been conducted by MRE researchers. In this study, an investigation on the thermal and rheological properties of epoxidized natural rubber (ENR)-based MREs was performed. Various percentages of carbonyl iron particles (CIPs) were blended with the ENR compound using a two roll-mill for the preparation of the ENR-based MRE samples. The morphological, elemental, and thermal analyses were performed before the rheological test. Several characterizations, as well as the effects of the strain amplitude, temperature, and magnetic field on the rheological properties of ENR-based MRE samples, were evaluated. The micrographs and elemental results were well-correlated regarding the CIP and Fe contents, and a uniform distribution of CIPs was achieved. The results of the thermal test indicated that the incorporation of CIPs enhanced the thermal stability of the ENR-based MREs. Based on the rheological analysis, the storage modulus and loss factor were dependent on the CIP content and strain amplitude. The effect of temperature on the rheological properties revealed that the stiffness of the ENR-based MREs was considered stable, and they were appropriate to be employed in the MRE devices exposed to high temperatures above 45 °C.

## 1. Introduction

Magnetorheological (MR) materials are classified as smart materials while the rheological and mechanical properties can be controlled by altering the magnetic fields. These smart materials have been challenging researchers for many years since their development offered vast possibilities for base material selection. Among other types of MR materials such as MR fluid, MR gel, and MR grease, MR elastomers (MREs) have become a hot topic in MR research due to the high stability and compatibility between the matrix and magnetizable particles [1,2,3,4]. This fact prevents particle sedimentation and leakage issues, which frequently occur when other MR materials are utilized. MRE has unique factors corresponding to field-dependent rheological and mechanical properties that have been investigated extensively [1,2,3,4,5,6,7,8]. In the past decade, these properties contribute to large promising applications for MREs particularly in three main areas known as: (a) vibration and noise control, (b) sensing devices, and (c) actuators [9,10,11,12,13,14,15,16,17,18]. Recently, these intelligent materials have been penetrated in a biomedical engineering field where MRE has been utilized as a soft material for a magnetic-elastic soft millimeter-scale robot [19,20]. Controllable rheological properties of MRE has not only overcome an issue of the small-scale robot corresponding to mobility limitation due to different material or texture in an unstructured environment. MRE makes the soft robot to be able to transit reversibly between different liquid and solid terrains, and switched between locomotive modes, as extensively described by Hu et al. [19].

It is acknowledged that MREs are considered to be viscoelastic composites that are composed of two main elements, which is the elastomer matrix and magnetizable particles. Additionally, the two types of MREs are referred as isotropic and anisotropic. The isotropic and anisotropic MREs have a uniform and aligned distribution of magnetizable particles, respectively. The magneto-induced storage modulus (stiffness) and loss factor (damping) as well as MR effect are the essential parameters that need to be taken into consideration in achieving desirable MRE properties that suit many types of applications. Many efforts have been made toward investigating and improving the performance of MREs. For example, there are attempts to use a variety of elastomers and magnetizable materials such as saturated and unsaturated rubbers and soft and hard magnetizable particles, respectively [2,7,21,22,23,24,25,26,27,28,29]. Ubaidillah et al. [11] reported that saturated elastomers, particularly silicone rubber (SiR), have often been studied as MRE matrices, and they contributed up to 51.5% in total due to the simplicity of fabrication. SiR-based MREs demonstrated a high MR effect because of the enormous difference between the initial and maximum magneto-induced storage modulus [8,30]. However, the SiR-based MREs exhibited a low magneto-induced storage modulus and, thus, could only be implemented in specific applications. Additionally, SiR is a petroleum-based rubber that is categorized as unfriendly to the environment and could lead to the growth of global warming [2,31]. In this case, it is apparent that the selection of matrix materials affects the production of the MREs that have anticipated controllable stiffness and damping properties. Consequently, this becomes one of the most challenging parts of this research area.

Therefore, epoxidized natural rubber (ENR) was chosen in this study since it is well-known as an environmentally-friendly or green material made of the natural resource corresponding to natural rubber (NR) [2,32,33,34,35,36]. Fundamentally, the ENR is synthesized by modifying NR latex via an epoxidation process. The epoxidation process refers to the mixing of a chemical compound known as hydrogen peroxide with NR latex, which consequently transforms the carbon–carbon double bonds of NR latex into an epoxy group. Through this process, the compatibility with polar polymers, wear, thermal, and mechanical properties are improved [36]. Furthermore, as a new rubber, the ENR not only has better properties than NR such as high thermal resistance but some features of synthetic rubber could be attained [33,34,36,37,38]. The ENR has been commercialized as a ‘green tyre’ [38,39]. However, more research studies have been discovered recently on its usage due to its favorable storage and loss modulus as well as environmentally-friendly characteristics such as compatibilizer and impact modifier [32,37,40,41,42]. As the previously mentioned, ENR has a set of properties that are favorable for MREs and, particularly, its thermal resistance. Nevertheless, research reports on the utilization of ENR as an MRE matrix are still rarely found [2,4,43]. Wang et al. [43] is considered as a pioneer in finding the use of ENR as MRE matrix while they employed a mixture of polychloroprene rubber and ENR. They discovered that this rubber blend had a good interfacial adhesion between the magnetizable particles and contributed to the excellent rheological properties. Khairi et al. [4] examined the effect of plasticizer on the field-dependent rheological properties of ENR-based MRE. The investigations concerning the utilization of the ENR in the MRE research area still has a wide scope. A comprehensive assessment on ENR-based MRE based as a single matrix would be an interesting step.

Previously, we prepared the isotropic MREs using ENR alone as an MRE matrix using conventional rubber processing [2]. The results also showed that ENR could be a promising alternative material for an MRE elastomer matrix without blending it with other types of rubber. Yet, the characterization tests, mainly thermal analysis and its correlation with the field-dependent rheological properties, have not been discussed in the previous studies. Yet, the thermal characterization is crucial in order to implement the MRE in any MRE devices. This is because MRE devices commonly operate in particular conditions, which involve a wide range of frequency, strain loadings, magnetic fields, and temperatures. For instance, Yu et al. [44] investigated the effect of temperature of the performance of the MRE isolator and found that the maximum attenuation of the stiffness and damping can reach 26.42% and 34.55%, respectively, with the temperature increasing from 30 to 80 °C. As previously mentioned, we have performed an investigation on the storage modulus and loss factor of MRE-based ENR with different frequency loading and magnetic field. To extent our research on the use of ENR as a new matrix for MRE, the temperature and strain amplitude should be taken into consideration with respect to design and application of the MRE-based device. To the best of the authors’ knowledge, a paucity of thermal dependent analyses has been conducted, which involve different matrices. Wan et al. [45] examined the thermal effect of viscoelastic properties of anisotropic SiR-based MREs under compression loading. The results revealed that the storage modulus of SiR-based MREs decreased with an increasing temperature up to 50 °C and increased slightly or became constant beyond this temperature when exposed to 300 and 500 mT, respectively. Zhang et al. [46] utilized polybutadiene (BR) and natural rubbers (NR) as matrices. The results were well-agreed with DSC results where they found that the storage modulus linearly diminished with temperature in case of BR. Meanwhile, the storage modulus declined linearly up to 50 °C and then increased slightly as the temperature increased to 70 °C, and then became slender over this temperature range. The MRE matrix is also one of the factors, which could significantly affect the thermal stability of MRE. Despite matrices, the numerical mathematical models have been developed by several researchers to study the effect of temperature on MRE that agree with the law of thermodynamics [47,48,49,50]. Mathematical modeling of large strain deformation that was assumed to occur due to mechanical viscoelastic effects and resistance of MRE towards magnetization has been discovered by Saxena et al. [49]. Due to this matter, the determination of the strain-dependent properties, thermal stability, and durability of the MREs is of paramount importance, especially concerning MRE applications. Moreover, the understanding of various parameters of thermal degradation is essential to develop a rational technology for polymer and composite processing as well as high-temperature applications.

Therefore, this study aims to investigate the thermal characteristics of ENR-based MREs and the correlation between the thermal and rheological properties. Additionally, the particle size, morphological analyses, and elemental analyses were also performed experimentally. The ENR-based MRE samples were fabricated by blending the ENR compound with carbonyl iron particles (CIPs) at various weight percentages. The thermal characterizations were carried out via thermal gravimetric analysis and differential scanning calorimetry. For rheological measurements, the effects of the strain amplitude and temperature on the rheological properties of the ENR-based MREs were analyzed through dynamic shear measurement using a rotational rheometer. 

## 2. Results and Discussions

### 2.1. Particle Size

**Figure 1:**
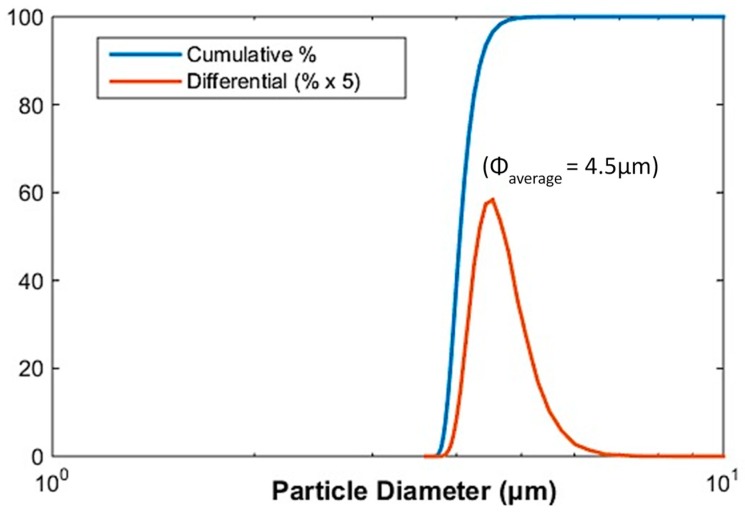
Particle size distribution of carbonyl iron particles (CIPs).

Figure 1 presents the results of the particle size distributions of the CIPs. The average diameter (Φ_average_) of the CIPs was determined to be 4.5 μm (labeled as a dotted line), with a size distribution of *d10* = 2.6 μm, *d50* = 4.5 μm, and *d90* = 7.5 μm, as shown in Figure 1. The particle size distribution was measured since the performance of the MREs is dependent on the size and distribution of particles in the elastomer matrix, specifically when subjected to a uniform magnetic field.

### 2.2. Microstructure

**Figure 2:**
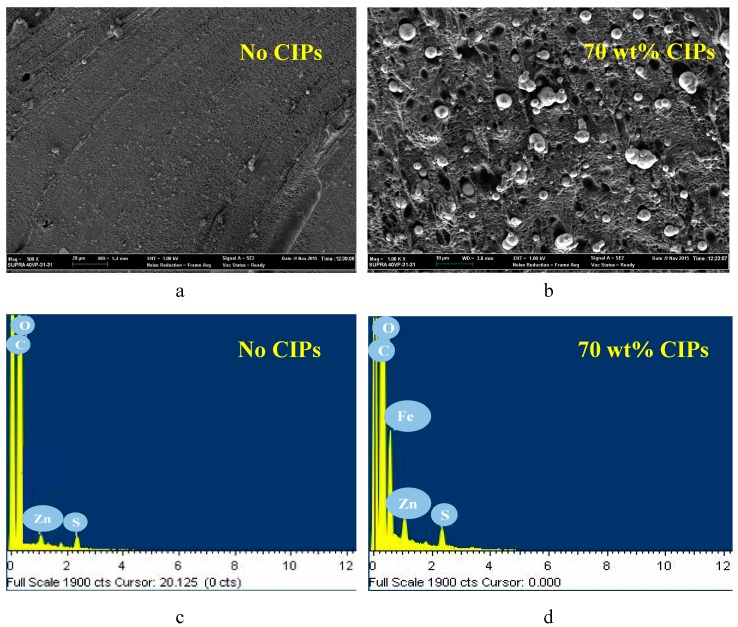
Micrographs and EDX graphs of samples with (**a**) and (**c**) no CIPs, and (**b**) and (**d**) 70 wt % of CIPs at 1000× magnification.

Figure 2a–d depicts the micrographs and energy dispersive X-ray spectroscopy (EDX) graphs of the samples containing 0 wt % and 70 wt % CIPs.

Based on Figure 2a, it is observed that no CIPs were available in the sample, and the ENR matrix was accessible in black color. On the other hand, CIPs, which as spherical in white and gray, were uniformly disseminated in the ENR matrix, as shown in Figure 2b. The micrographs confirmed that the fabricated ENR-based MREs were of the isotropic type. The EDX analysis was performed on the same micrograph to justify the presence of CIPs as a spherical shape that was distributed in the ENR matrix. As shown in Figure 2c,d, the existence of CIPs was exhibited in the form of the element iron (Fe) since they contain ≥97% Fe. It is proven that no CIPs were detected in the sample without CIPs since no peak corresponding to Fe was observed in Figure 2c. Meanwhile, there was a sharp peak of the element Fe in the MRE sample, which is comprised of 70 wt % CIPs, as identified in Figure 2d. Hence, the results of micrographs were well-correlated with EDX graphs for ENR-based MRE samples. Other than element Fe, several peaks that referred to carbon (C), sulfur (S), oxygen (O), and zinc (Z) were also seen in the EDX graphs.

Table 1 details the elemental results of the ENR-based MREs for samples with various CIP contents. 0 wt % of the element Fe was detected for the sample without CIPs and increasing the CIP content resulted in greater values of the element Fe. The Fe increase was in the range of 4.36–8.26 wt % since the amount of CIPs increased 10 to 70 wt %. Similar to the EDX results, carbon, sulfur, zinc, and oxygen were detected in each sample. In fact, a higher content of carbon compared to other elements was revealed since a high amount of carbon black was added during the preparation of the rubber compound to improve the bonding between the ENR matrix and CIPs [51,52]. As a highlight, it is apparent that the micrographs and elemental results of ENR-based MREs correlate well and demonstrate the presence of CIPs distributed in the ENR matrix.

### 2.3. Thermal Characteristics

#### 2.3.1. Thermal Gravimetric

**Figure 3:**
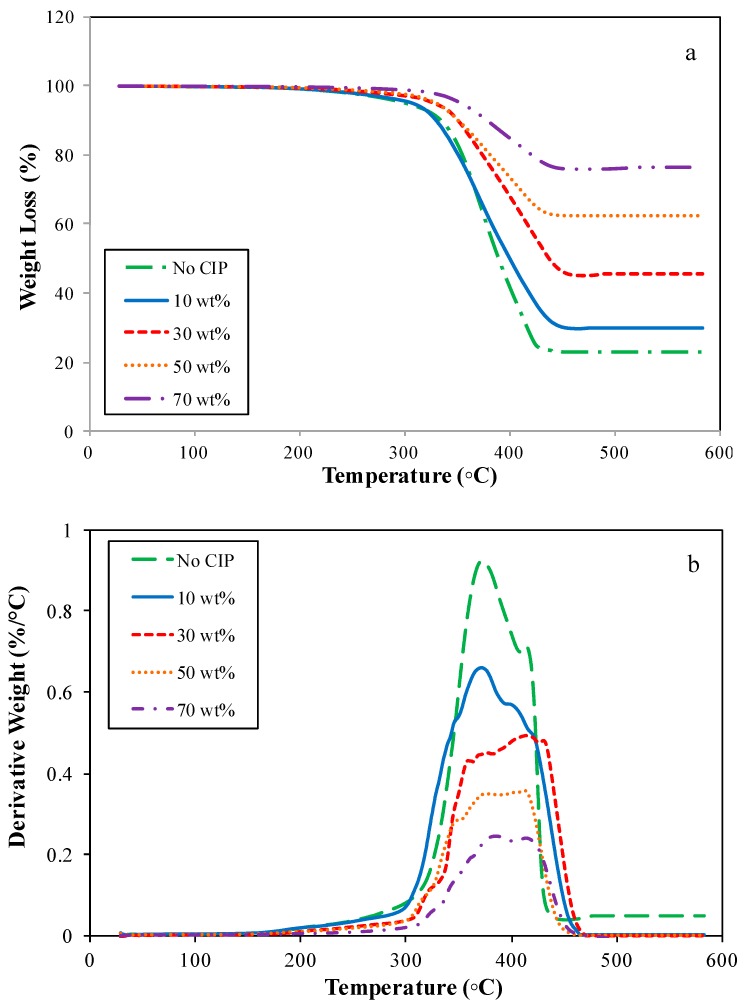
Thermal features of ENR-based MREs. (**a**) Thermogravimetric (TG) curves and (**b**) Differential thermal gravimetric (DTG) curves.

The thermal properties of ENR-based MREs for various CIP contents in a nitrogen atmosphere as well as the characteristics of the thermal decomposition are demonstrated in Figure 3 and Table 2, respectively. Figure 3a,b displays the thermal gravimetric (TG) and differential thermal gravimetric (DTG) curves of the ENR-based MREs for all of the CIP contents, respectively.

According to the TG curves, two decomposition stages occur ranging from 250 to 332 °C and 315 to 463 °C. At the initial decomposition (stage I) of all samples, it is apparent that the TG curves declined slightly, and no peak was observed in the DTG curves, as illustrated in Figure 3. The stage I was attributed to the decomposition of volatile matter comprised of certain additives that were added to the samples during the compounding process. Meanwhile, a dramatic decrease of the TG patterns during the second stage was observed, and the values of the residue at 600 °C are available in Table 2. Moreover, the second thermal decomposition (stage II) was accompanied by the DTG peaks shown in Figure 3b in a similar temperature region. These results demonstrated that the thermal decomposition of the ENR-based MREs exhibits two-step-degradation. Stage II designated a polymer decomposition whereby the ENR matrix decomposed in this temperature range. In general, the mass loss of the ENR-based MREs was predominantly because of ENR matrix decomposition since the CIPs only started to decompose at temperatures beyond 1000 °C [23,53,54]. Lee et al. [36] have conducted several analysis on the ENR and NR-ENR blends. They found that ENR started to decompose at a higher temperature when compared to NR. It is noted that the ENR provided better thermal resistance, which consequently improved when it was mixed with CIPs.

Based on Figure 3b, the endothermic peaks in the DTG curves were observed in a temperature region of approximately 300 to 460 °C, whereby the stage II of decomposition occurred as mentioned previously. The peaks imply the breaking of the cross-linked polymer chains or networks depending on the amount of ENR matrix present in the sample. Thus, the broader peaks that were observed by increasing the CIP content corresponded to a decrease of the cross-linked polymer networks. 

Table 2 demonstrates the thermal decomposition temperatures of ENR-based MREs for various wt % of CIPs. In Table 2, *T*_onset_ and *T*_end_ denote the temperatures wherein the decomposition of the ENR matrix starts and ends, and *T*_max1_ and *T*_max2_ are referred to as the temperatures for the maximum thermal decomposition rates at stages I and II.

The results indicated that the polymer decomposition of the ENR-based MRE with the increase of CIPs started to occur at a high temperature since *T*_onset_ exhibited an increasing trend. The *T*_onset_ increased from 250 to 276 °C when the CIP content increased from 0 to 70 wt %. Moreover, the *T*_max1_, the *T*_max2_, and the *T*_end_ of the samples showed similar trends in which all of the parameters increased with the addition of CIPs, which was accompanied by increasing the residue from 23.18% to 76.36%. Apparently, the addition of CIPs delayed the decomposition process because the CIPs restrained the mobility of the ENR molecular chains. This could also be due to the difficulty of destructing the crosslinked polymer chains since CIPs occupied the voids and became barriers, which leads to a slow decomposition process. It can be deduced that ENR and the addition of CIPs provides better thermal resistance of MRE corresponding to both the TG-DTG and DSC outcomes.

The earlier reports also implied that the addition of magnetic particles contributed to an improvement in thermal stability of MRE. For instance, Gong et al. [55] investigated the use of Pu MR foam as the matrix and CIPs as magnetic particles. The results showed that, with the incorporation of CIPs, the *T*_onset_ and *T*_max1_ increased because CIPs restrain the mobility of the PU molecular chain, which delayed the decomposition of MRE. Venkatanarasimhan and Raghavachari [56] also obtained the same results in which utilization of magnetite nanoparticles into NR and ENR contributed to improvement in thermal stability. In fact, ENR enhanced the thermal stability of the samples better than NR with higher values of 10% weight decomposition temperature (*T*_10%_). Moreover, Chuayjuljit et al. [57] found that the thermal stability of NR was enhanced by introducing the epoxide groups onto its backbones, with the *T*_onset_, T_50%_, and *T*_max_ of ENR were shifted to higher temperatures than those for NR. This is because the epoxide groups enlarged the intermolecular attraction and diminished the chain mobility. Then the thermal stability was improved. Wang et al. [43] found the similar pattern and concluded that the lower weight loss that is characteristic of a higher iron particle content exhibited better thermal stability. As shown in Table 2, a lower content of CIPs implies a higher weight reduction of the samples, whereby the residues of the samples increased from 28.18% to 76.16%. Yet, Wu et al. [53] reported that the magnetic particles greatly gave an adverse effect on thermal stability of PU-based MREs with a decreasing of *T*_onset_ and *T*_max1_. Furthermore, the anisotropic type enhanced the thermal conductivity and diminished the thermal stability of PU-based MREs compared to the isotropic one due to aligned chain-like structure of CIPs in PU, which acted as a pathway for heat transfer. 

#### 2.3.2. Differential Scanning Calorimetry

**Figure 4:**
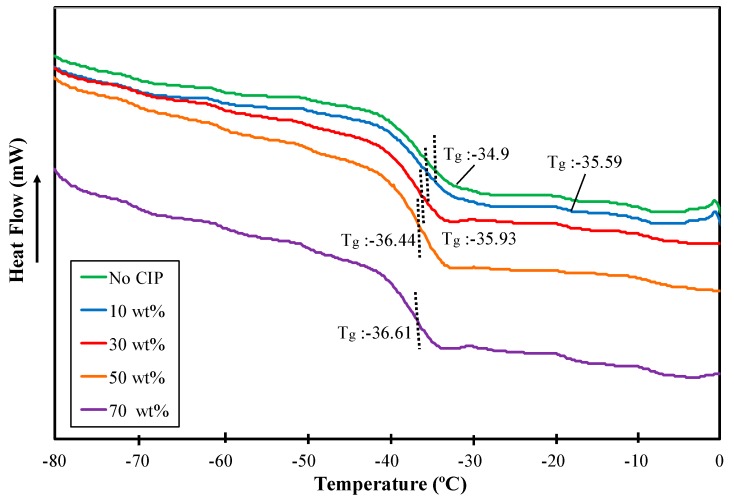
Differential Scanning Calorimetry curves for ENR-based MREs for different CIPs loading.

Figure 4 presents the differential scanning calorimetry (DSC) curves for ENR-based MRE samples together with the glass transition temperature values (*T_g_*) for different CIP loadings.

In this scenario, *T_g_* refers to a temperature region (labeled as a dotted line) where the polymer transforms from a hard and glassy state to a soft and/or rubbery state. The DSC curves for samples with and without CIPs exhibited an endothermic heat flow, which was accompanied by an exothermic peak in a temperature region of −30 to −43 °C. The endothermic peak shifted toward a low temperature as the CIP content increased, as shown in Figure 4. The *T_g_* regions of the ENR-based MREs were obtained at a temperature between −35 to −42 °C due to phase transformation. Similarly, the *T_g_* also shifted toward negative values. The values are −34.9 to −36.61 °C with increasing CIP contents from 0 to 70 wt %. In the case of all samples, the *T_g_* was shifted insignificantly in the range of 0.69 to 1.71 °C. This is probably due to mitigation of the degree of crystallinity of the ENR-based MRE samples [58,59]. Additionally, as the CIP content increased, the CIPs became entrapped between the polymer chains and increased the free volume of the polymer chains. Since the availability of the free volume of polymer chains increased, the polymer became quickly transformed to a rubbery state compared to samples without CIPs and that led to a reduction of the *T_g_*. Therefore, these results signified that the insertion of CIPs contributed to an insignificant change in the *T_g_.*

Nevertheless, these outcomes were correlated with previous research reported by Sun et al. [60] in which the *T_g_* value was decreased by the addition of magnetic particles in case of both types of MRE, isotropic, and anisotropic. In fact, the isotropic polybutadiene rubber-based MRE had a smaller change of *T_g_* values, which, in contrast to the anisotropic one, gave a large effect on *T_g_* values with the addition of particles. This was due to the mitigation of polymer chains’ free volume caused by the column or an aligned structure of magnetic particles that existed in anisotropic MRE. Wu et al. [24] obtained the same results using isotropic PU-based MRE. On the other hand, the decrement of the *T_g_* values reported by those MRE researchers was large (7 to 9 °C). Hence, the addition of magnetic particles need to be taken into consideration since it significantly influenced the phase transition region. Meanwhile, ENR does not largely affect the *T_g_*, which suggests that ENR-based MRE could be widely employed pertaining to its service temperature. The DSC results of other types of MRE matrix such as NR and SiR, though, have not been reported. 

### 2.4. Rheological Properties

#### 2.4.1. Strain Sweep

It is well agreed upon that viscoelastic materials are usually subjected to shear deformation due to their intrinsic properties. When they are subjected to an oscillation load, some part of the energy is stored temporarily and can be recovered after each cycle. The rest of the energy is dissipated as heat, and this kind of energy cannot be recovered. The ability to store the energy temporarily is referred as stiffness and acknowledged as the storage modulus. In the meantime, the viscous properties or loss modulus represents the capability of dissipating the deformation energy in the form of heat. Moreover, the loss factor is referred to as a damping property of the MRE material, where the value is obtained by dividing the loss modulus by the storage modulus. Fundamentally, the storage modulus and loss factor are parameters that designate the rheological properties of MREs. Selected plots will be discussed thoroughly for strain and temperature sweep tests because the patterns of the graphs were similar.

**Figure 5:**
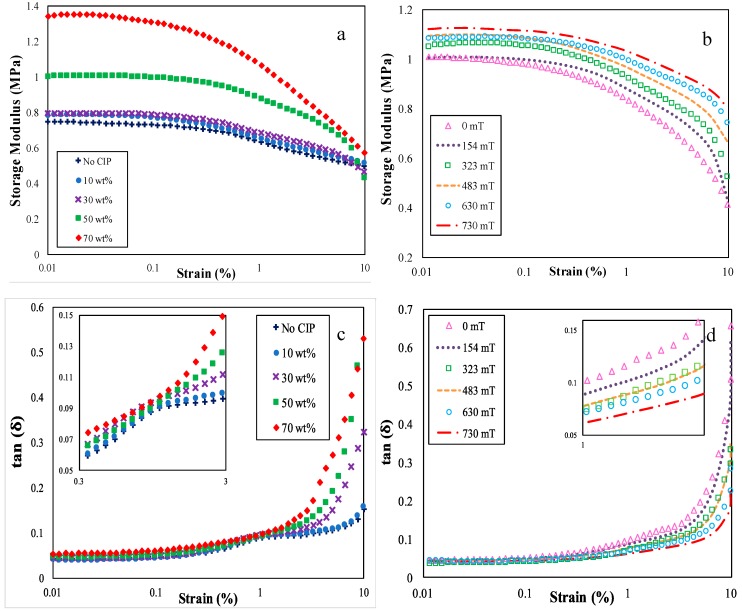
Results of (**a**,**b**) storage modulus, and (**c**,**d**) loss factor of ENR-based MREs at different CIP content and magnetic fields for various strain amplitudes.

Figure 5 depicts the influence of the strain amplitude for various CIP contents and magnetic fields on the storage modulus and loss factor of ENR-based MREs, which were obtained for MRE samples that were subjected to 323 mT and 30 wt % of CIPs, respectively. As referred to in Figure 5a and Figure 5b, a decreasing trend was observed for all CIP loadings and applied magnetic fields in such a way that the storage modulus steadily decreased as the strain amplitude increased from approximately 0.01% to 1%, and it remarkably diminished beyond these values up to 10%. In contrast, the loss factor was increased with the increase of the strain amplitude. The increase of the loss factor was also composed of two stages. It was increased slightly at approximately 0.01% to 0.85% and then dramatically increased over this strain amplitude range.

These results implied that the ENR-based MREs were strain-dependent materials. Similar to other viscoelastic materials, the steady decrease and increase of the storage and loss factors in the mentioned range of strain amplitudes signified the linear viscoelastic region. The determination of the linear viscoelastic (LVE) region of ENR-based MRE followed the Agire-Olabide’s method [11]. At this point, the shear strain did not have a significant influence on the network structure or cross-linking of polymer in the ENR-based MRE samples. The subsequent region of dramatic decreases and increases in the storage modulus and loss factor, respectively, which corresponded to a non-linear viscoelastic region of ENR-based MRE samples. In this region, the structure of the polymer network in the samples started to be disrupted by the applied shear strain, and MRE materials became more viscous rather than the elastic solid. Therefore, the storage modulus was diminished while the loss factor was increased with increasing strain amplitude. Thus, it is noteworthy to determine these regions in order to prevent analyzing the frequency, temperature, and magnetic field sweep tests in the region where the structure was already damaged. Therefore, the viscoelastic region of ENR-based MRE samples was determined to be up to approximately 1% of the strain amplitude. Noticeably, the same value of the linear viscoelastic region was measured regardless of the magnetic field and CIP content. These results revealed that the linear viscoelastic region for 10 to 70 wt % CIPs of the ENR-based MREs was stable and referable. These patterns followed the characteristics of viscoelastic materials, which was due to the Payne effect that was discovered by several researchers [8,61,62,63].

Furthermore, these outcomes were also well-agreed with studies reported by numerous researchers [5,7,22,26,64,65,66,67,68,69]. They discovered that the storage modulus and loss factor were augmented with increasing CIPs content. It could be said that the addition of CIPs content in MRE provide enhancement on dipole-dipole or interparticle magnetic forces and interfacial slipping among adjacent CIPs and rubber matrix since the distance among particles became closer. Khimi et al. [64] and Li et al. [22] used NR and silicon rubber as the matrix, respectively. Although, the values of storage modulus increased with particles content, the values were lower than using ENR in this study. Thus, ENR has a better ability to store dissipated energy during the deformation than those rubbers and these abilities could be improved by the addition of CIPs content. 

In case of both parameters, a similar graph pattern was observed in which the values of the storage modulus and loss factor increased with the addition of CIPs in the ENR-based MRE samples and rise of the applied magnetic fields, respectively. The increase of the maximum storage modulus with the increase of the applied magnetic fields was approximately 0.1 MPa, while the loss tangent was approximately 0.01 MPa, as depicted in Figure 5b,d. Increasing the applied magnetic fields resulted in the enhancement of interparticle magnetic forces among CIPs as well as dissipated energy in the ENR-based MREs. In contrast, Figure 5a shows that the storage modulus was improved marginally when the CIP content rose from 10 to 30 wt %, and the increase was below 0.1 MPa. Afterward, the storage modulus was evidently augmented when the CIPs increased from 50 to 70 wt %. The rise of the maximum storage modulus in the case of 10 to 70 wt % CIPs was approximately 0.8 to 1.37 MPa. Additionally, the increase of the highest values of the loss factor, which were obtained at the maximum strain amplitude, was 0.19–0.35 MPa. Apparently, the increment of the magnetic field from 0 to 730 mT gave a small effect depending upon CIPs content. Therefore, it can be deduced that changes of these parameters were mainly influenced by CIPs content and the effect of the magnetic field was more pronounced by the addition of CIPs.

As previously mentioned, the CIPs content resulted in the enhancement of magnetic forces and friction caused by slipping among CIPs due to the reduction of the interparticle distance between CIPs. The results were in correlation with the magnetization results reported elsewhere [2], as the increment of CIPs led to an improvement of magnetic properties with respect to the strong magnetic forces among CIPs. In fact, the magnetic forces may resist the applied shear deformation as the particles become closer and generate friction among CIPs simultaneously. As reported by Chen et al. [70] and Fan et al. [71], stiffness and damping properties of MREs greatly depend on the distance and interfacial slipping or friction between the rubber matrix and magnetic particles. Hence, more energy was stored and dissipated within MRE samples as the rise of CIPs content. Increasing the storage modulus and loss factor basically showed that the MRE samples became stiffer, and the damping capability was enhanced as CIPs were added to the sample when subjected to an elevated strain deformation. Yet, an increment of the storage modulus was more prominent when compared to the loss factor, which shows that magnetic forces dominate the properties of ENR-based MRE samples. The wider range of stiffness and damping properties is beneficial for applications that were exposed to low strain amplitude (below than 0.4%) such as shock absorber.

#### 2.4.2. Temperature Sweep

**Figure 6:**
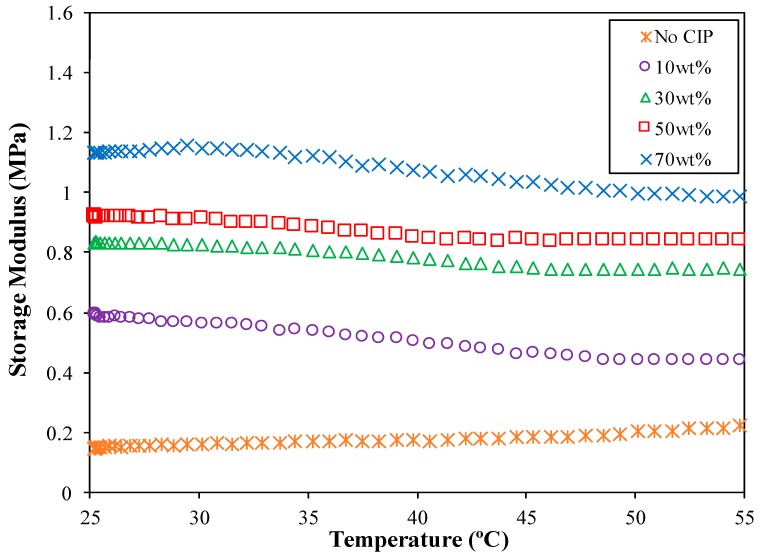
The influence of temperature on storage modulus of the ENR-based MREs at 1A for different CIPs content.

The temperature influence on the shear storage modulus of the ENR-based MREs was evaluated in this section. Figure 6 illustrates the influence of temperature on the shear storage modulus of the ENR-based MREs at 1A for different CIP contents. 

Apparently, as increasing temperatures were applied to the MRE, the values of the shear storage modulus showed two different trends, as illustrated in Figure 6. They first declined and then remained at nearly steady values when the testing temperature increased. In other words, the shear storage modulus of ENR-based MREs that contained 10 to 70 wt % CIPs was marginally diminished as the temperature increased up to 45 °C. Afterward, the effect of the temperature was not so clear with a further increase in temperature. Conversely, the shear storage modulus for samples without CIPs showed a temperature-dependent characteristic in which it increased steadily from 0.17 to 2.1 MPa with an increasing temperature. It is, however, the increment was only 0.05 to 0.1MPa since the temperature increased from 25 to 55 °C. To the best of the authors’ knowledge, these unique findings has not been reported elsewhere. The result is likely related to cross-linked monomer and filler within the ENR in which further analysis is required such as increasing temperature analysis of more than 55 °C. The effect of temperature on the storage modulus of ENR beyond this temperature value might be useful in identifying the cause of the phenomena.

Based on Figure 6, it shows that the CIP content certainly affect the thermal behavior of ENR-based MRE. These results revealed that the stiffness of the ENR-based MREs was considered stable under a high temperature above 45 °C due to the formation of a new balance between the particle mobility and interaction, and the effect of the attractive forces is not noticeable [33,67]. Hence, the ENR-based MREs are considered appropriate to employ in the MRE devices exposed to temperatures higher than 45 °C, as they are not influenced by the temperature [46].

Additionally, the CIP content also exhibited an essential effect on the storage modulus of ENR-based MREs under various temperatures. The higher the CIP loading, the higher the storage modulus that was obtained. This may be due to the rise of friction between CIPs and the ENR matrix generated from the ease of mobility of CIPs. At temperatures lower than 45 °C, the magneto-induced storage modulus, which also corresponds to the stiffness of ENR-based MREs, was mainly decided by the magnetic forces of crosslinked polymer chains and the mobility of the CIPs. However, the magnetic forces between CIPs are primarily caused by the magnetic interactions between iron particles, and the temperature has nearly no influence on magnetic interactions. Therefore, the storage modulus barely changed when the temperatures increased. 

**Figure 7:**
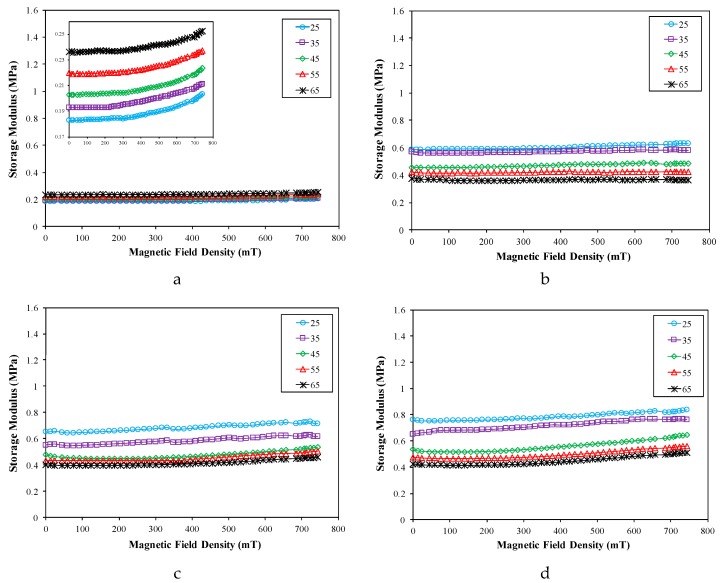

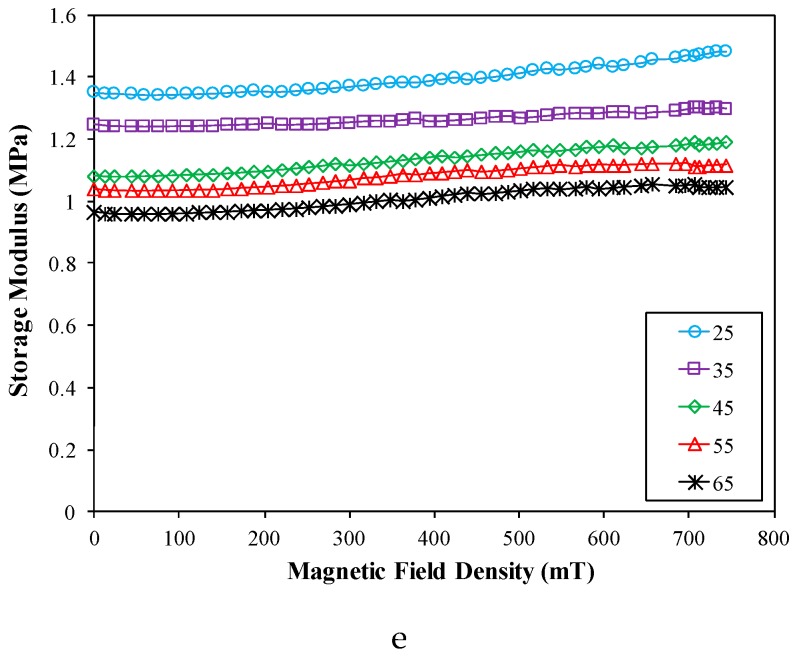
The storage modulus of the ENR-based MREs: (**a**) 0 wt %, (**b**) 10 wt %, (**c**) 30 wt %, (**d**) 50 wt %, and (**e**) 70 wt % of CIPs.

For further analysis of the effect of temperature, a constant temperature starting from 25 to 65 °C with an increase of 10 °C was applied in an elevated magnetic field. Figure 7 depicts the effect of temperature on the magneto-induced storage modulus of the ENR-based MREs for all CIP contents at various magnetic field densities.

Based on these figures, it is apparent that the magneto-induced storage modulus of ENR-based MREs showed a similar pattern, which slightly increased with rises in the magnetic field density when the MRE samples were subjected to a constant temperature. In other words, the storage modulus of the MRE declines with increasing temperature under the same magnetic field, which is also a common characteristic of polymers. Furthermore, the influence of temperature on the magneto-induced storage modulus was clearly equivalent and agreed well with the discussion related to Figure 6. The magneto-induced storage modulus marginally diminished with the rise in temperature from 25 to 45 °C, and, beyond this temperature range, a steady decrease of this parameter was observed. Therefore, it can be concluded that the temperature influenced the magneto-induced storage modulus at temperatures below 45 °C, and, above this temperature, the effect becomes almost negligible, which correlates with the previous results. In addition, the magneto-induced storage modulus for samples without CIPs exhibited a temperature-dependent property in which it increased steadily with temperature, which also correlates with the results illustrated in Figure 6. These outcomes are attributed to the effect of the crosslinked polymer chain, the mobility of CIPs, and magnetic forces among CIPs. In the presence of a magnetic field, a magnetic interaction between the CIPs was formed, and the shear storage modulus of MRE samples was decided by the matrix and filler particles.

The results were in agreement with outcomes obtained by Zhang et al. [46] and Xu et al. [67]. They used NR and PU as the matrix and the results revealed that the magneto-induced storage modulus was decreased at up to 40 and 50 °C, accordingly. Subsequently, the storage modulus became almost independent on the temperature beyond these values of temperatures. Similarly, Xuan et al. [72] prepared magnetic plasticine as the MRE matrix by mixing paraffin wax and petroleum jelly. They discovered that the magneto-induced storage modulus was diminished as the temperature decreased from 30 to 50 °C, and, beyond this temperature, the effect of temperature became negligible. They identified that the modulus of the viscoelastic materials corresponding to MREs is stable when the temperature is relatively high, whereby the modulus was changed with the temperature but kept stable when the temperature is higher than the critical point. Therefore, the service temperature of the ENR-based MRE was determined to be higher than 45 °C because the effect of the temperature is nearly negligible.

## 3. Experimental

### 3.1. MRE Fabrication

ENR-based MRE samples were fabricated conventionally using a two-roll mill and rubber curing equipment for the mixing and vulcanization processes, respectively. The ENR that contains 25 mol% of epoxidation was obtained from the Malaysian Rubber Development Corporation Berhad (MARDEC Berhad, Kuala Lumpur, Malaysia). Meanwhile, type C3518 CIPs, which consist of ≥97% iron, were acquired from Sigma-Aldrich, Steinheim am Albuch, Germany. The density of the CIPs is 7.86 g/mL, and the Mooney viscosity of ENR 25 is 110.

The fabrication of ENR-based MRE samples was described elsewhere in our previous report [18], which was started by producing uncured ENR and followed by the subsequent addition of CIPs into the compound. The recipe of the ENR compound is as follows: 100 phr rubber, 2 phr stearic acid, 5 phr zinc oxide (ZnO), 19 phr carbon black type FEF N550, 5 phr aromatic oil, 2.3 phr sulfur, and 0.8 phr *N*-cyclo-hexyl-2-benzothiazole sulfenamides (CBS). The mass fractions of the CIPs in the matrix were 0, 10, 30, 50, and 70 wt %.

### 3.2. Particle Size Analysis

The particle size distributions of the carbonyl iron powders were measured experimentally by laser light scattering using a particle size analyzer (PSA) instrument (Shimadzu SALD-2300, Kuala Lumpur, Malaysia). 

### 3.3. Morphological Analysis

Morphological analysis of the samples was carried out via field emission scanning electron microscopy (FESEM, Supra 40VP-31-31, Bangi, Malaysia). The micrographs of MRE samples were performed on the sample cross-section at 1000× magnifications using a low voltage acceleration of 1 kV. The samples were not covered with any coatings since they were already conductive. Furthermore, the CIP distribution within the ENR matrix and elemental analysis were characterized using energy dispersive X-ray spectroscopy (EDX) with mapping image analysis.

### 3.4. Thermal Properties

#### 3.4.1. Thermal Gravimetric Analysis

The thermal gravimetric analysis (TGA) of ENR-based MRE samples was conducted using a Thermal Gravimetric Analyzer Q50, TA Instruments (Kuala Lumpur, Malaysia). The mass of MRE specimens was measured to be approximately in the range of 8 to 10 mg. The MRE specimens were heated from an ambient temperature of approximately 25 to 600 °C under a constant heating rate of 10 °C/min in a nitrogen gas environment (20 mL/min flow rate). 

#### 3.4.2. Differential Scanning Calorimetry

The differential scanning calorimetry (DSC) examination required a DSC-60 apparatus (Shimadzu, Japan). The MRE samples were cooled from an ambient temperature to −100 °C using liquid nitrogen, which was followed by heating to 0 °C. The convection cooling rate was set at 10 °C/min.

### 3.5. Rheological Measurements

**Figure 8:**
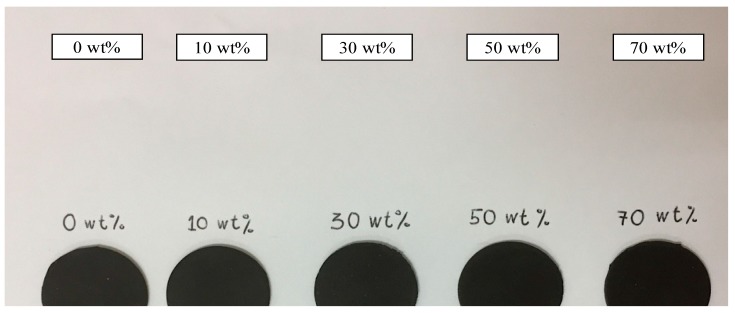
The cylindrical film of ENR-based MRE samples prepared for rheological measurement.

The rheological tests consisted of evaluating the strain, and temperature sweeps were performed using a rotational rheometer (MCR 302, Anton Paar Company, Germany) with a Magnetorheological Device (MRD) 70 as the shear probe. The MRD produced magnetic fields, which flow perpendicularly (90°) to the shear movement. A temperature control device (Viscotherm VT2, Anton Paar, Germany) was used to amend the measuring temperature. The MRE samples were cut into a cylindrical film with dimensions of 20 mm × 1 mm, as shown in Figure 8. The samples were placed between a rotary disk and a parallel base plate. In this study, an oscillatory shear was used for all of the rheological experiments.

For strain sweep measurements, the strain amplitude was varied from 0.01% to 10%. Meanwhile, the frequency was kept constant at 1 Hz, and the current was increased from 0 to 5 A by an increment of 1 A at each measurement. For the temperature sweep test, the effect of temperature on the storage modulus was investigated by varying the temperature from 25 to 65 °C for all of the samples under application of the constant magnetic field. In addition, the temperature was increased by 10 °C at the elevated magnetic flux for further analysis. To analyze the effects of magnetic fields at various strain amplitudes and temperatures, the current was increased from 0 to 5 A by an increment of approximately 1 A at each measurement. The magnetic field values for each current were recorded using the Gauss meter. Instead of current, all data were plotted based on magnetic field values. The rheological measurements were conducted at room temperature.

## 4. Conclusions

The ENR was utilized as an MRE elastomer matrix, and the thermal characteristics, as well as the rheological properties of ENR-based MRE samples, were studied comprehensively. The micrograph of ENR-based MREs demonstrated that CIPs are homogeneously disseminated within the samples, which signifies that the samples are isotropic type MRE. The results of the thermal characterization test indicated that the ENR matrix and incorporation of CIPs provided better thermal resistance of MRE due to an increment of thermal decomposition temperature from 250 to 276 °C. The uniformly dispersed CIPs act as barriers and restrain the mobilization or destruction of the ENR network molecular chains, which consequently leads to a slow decomposition process. Based on the rheological data obtained, the storage modulus and loss factor were essentially dependent on the CIP content and strain amplitude, and they could be adjusted by altering the magnetic fields. The LVE regime of ENR-based MREs was determined to be less than 1% of the strain amplitude regardless of the CIP content and applied magnetic field. The results revealed that the stiffness value of the ENR-based MREs was considered to be stable and this sample is appropriate to be employed in the MRE devices commonly exposed to high temperatures above 45 °C. Indeed, the thermal characteristics and temperature-dependent rheological properties were necessary to be investigated prior to implementing the MRE in any device.

## Figures and Tables

**Table 1:** Elemental result of ENR-based MREs for various wt % of CIPs.

CIP Content (wt %)	Element (wt %)				
	Fe	C	S	Zn	O
0	0.00	84.24	1.51	1.42	12.83
10	0.96	83.11	1.26	1.60	13.07
30	6.89	80.26	1.53	2.12	9.20
50	11.25	74.64	1.24	2.17	10.70
70	19.51	70.57	1.46	1.73	6.73

**Table 2:** Thermal degradation temperatures of ENR-based MREs for various wt % of CIPs.

CIP Content (wt %)	*T*_onset_ (°C)	*T*_max1_ (°C)	*T*_max2_ (°C)	*T*_end_ (°C)	Residue at 600 (°C) (%)
0	250	300	380	420	23.18
10	251	308	388	448	30.15
30	260	320	397	450	45.31
50	265	327	404	455	62.61
70	276	332	409	463	76.36

## Abbreviations

MREs	Magnetorheological Elastomers
CIPs	Carbonyl Iron Particles
SiR	Silicone Rubber
NR	Natural Rubber
ENR	Epoxidized Natural Rubber
G’	Storage Modulus
Tan (δ)	Loss Factor
PSA	Particle Size Analyzer
FESEM	Field Emission Scanning Electron Microscope
EDX	Energy Dispersive X-ray Spectroscopy
TGA	Thermal Gravimetric Analysis
DTG	Differential Thermal Gravimetric
DSC	Differential Scanning Calorimetry
